# Solitary fibrous tumor of the scrotum: a case report and review of the literature

**DOI:** 10.1186/s12894-019-0573-2

**Published:** 2019-12-30

**Authors:** Tsung-Hsin Chang, Marcelo Chen, Chih-Chiao Lee

**Affiliations:** 1 Department of Urology, Mackay Memorial hospital, No.92, Sec. 2, Zhongshan N. Rd., Zhongshan Dist, 10449 Taipei City, Taiwan (Republic of China); 2 Mackay Medical College, No.46, Sec. 3, Zhongzheng Rd., Sanzhi Dist, 252 New Taipei City, Taiwan (Republic of China)

**Keywords:** Solitary fibrous tumor, Scrotum

## Abstract

**Background:**

Solitary fibrous tumor (SFT) is a rare soft tissue tumor originally reported in the pleura. Although it has been reported in various extra-pleural sites, the occurrence of SFT in the scrotum is extremely rare. Herein, we present a 48-year-old man who had scrotal SFT. There are very few reported cases of genitourinary SFTs, this is only the fifth report of SFT of the scrotum in the English medical literature.

**Case presentation:**

In this study, we report on a 48-year-old man who presented with a 5 × 8 cm scrotal mass between his testes. Physical examination revealed a 4.7 × 8.5 cm lobulated tumor mass located between his testicles. Surgical excision of the tumor with scrotal approach was done and pathology reported a SFT. The patient was alive without tumor recurrence or distant metastasis during ongoing follow-up for 9 months post-operatively..

**Conclusion:**

Scrotal SFTs are very rare and only five cases have been reported in English literature to date. Treatment often involves surgical resection, and a definite diagnosis is made with the help of immunohistochemistry. The current general consensus for the management of SFTs is long-term follow-up after surgical excision of the tumor.

## Background

Solitary fibrous tumor (SFT) is a rare mesenchymal spindle cell neoplasm usually originating from the pleura. It was first described by Klemperer and Rabin in 1931 [1] and has since been reported in various extra-pleural sites. However, reports of urogenital SFTs are extremely rare and only a few cases of scrotal SFTs have been reported [2–9]. Treatment usually involves enucleation and excision of the tumor. Diagnosis is made with the help of immunohistopathological examinations. We hereby report the clinical and pathological characteristics of scrotal SFT.

## Case presentation

A 48-year-old male presented with a slow-growing right scrotal mass for the past 2 years. This clearly-demarcated nodular mass was located over the middle-to-right side of the scrotum. The tumor had rapidly increased in size over the past 3 months, but there was no obvious pain or other symptoms. Physical examination revealed a 4.7 × 8.5 cm lobulated tumor mass located between his testicles, non adherent to the scrotum. It was freely movable with elastic consistency on palpation. The penis and testicles were normal in appearance. Testis tumor markers were all normal [alpha-fetoprotein (AFP) = 2.87 ng/mL, beta-human chorionic gonadotrophin (β-HCG) < 0.6 mIU/mL].

**Fig. 1:**
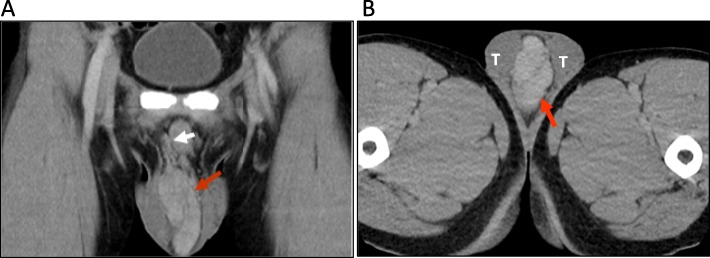
CT image demonstrating. (**a**) A 4.7 × 8.5 × 3.5 cm tumor (red arrow) with vascular supply (white arrow) arising from the right spermatic cord. (**b**) An extra-testicular mass (red arrow) within the scrotum between the two testes (T)

Scrotal ultrasonography showed a hypoechoic extra-testicular mass with clear contours and rich blood flow. Computed tomography (CT) showed a hypervascularized lobulated mass (4.7 × 8.5 cm) with contrast media enhancement in the midline of the scrotum (Fig. 1). He then underwent tumor excision via scrotal approach since the tumor did not seem originated from testicular or spermatic cord and the location of the tumor was superficial. A midline raphe incision was made and carry down to the tunica vaginalis. The tumor mass was not adherent to the surrounding tissues or testicles, it was a separated mass with clear margin that could be easily dissected with blunt dissection method. The tumor was completely excised after ligating the main feeding vessels.

**Fig. 2:**
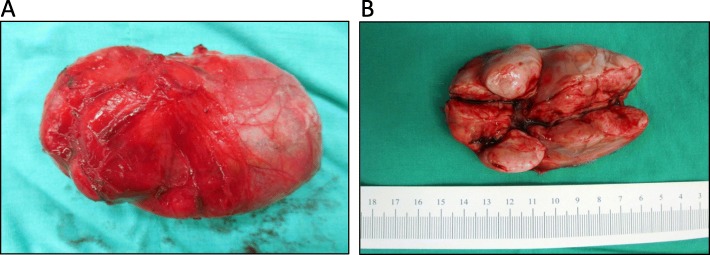
Gross pictures. (**a**) Gross appearance of the tumor showed three connected tumor nodules encapsulated within a fibrous capsule. (**b**) The cut section of the tumor showed three well-defined, lobulated, whitish firm tumors, measuring 7.5 × 6.3 × 3.5 cm in total size. Some mucinous components can also be seen

**Fig. 3:**
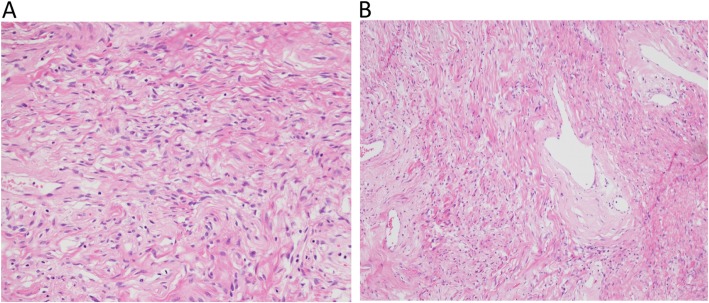
Hematoxylin and eosin staining demonstrating. (**a**) spindle cells with a fascicular growth pattern forming a “patternless” growth architecture. (**b**) Thin-walled, branched “staghorn” configuration of vessels can be seen

**Fig. 4:**
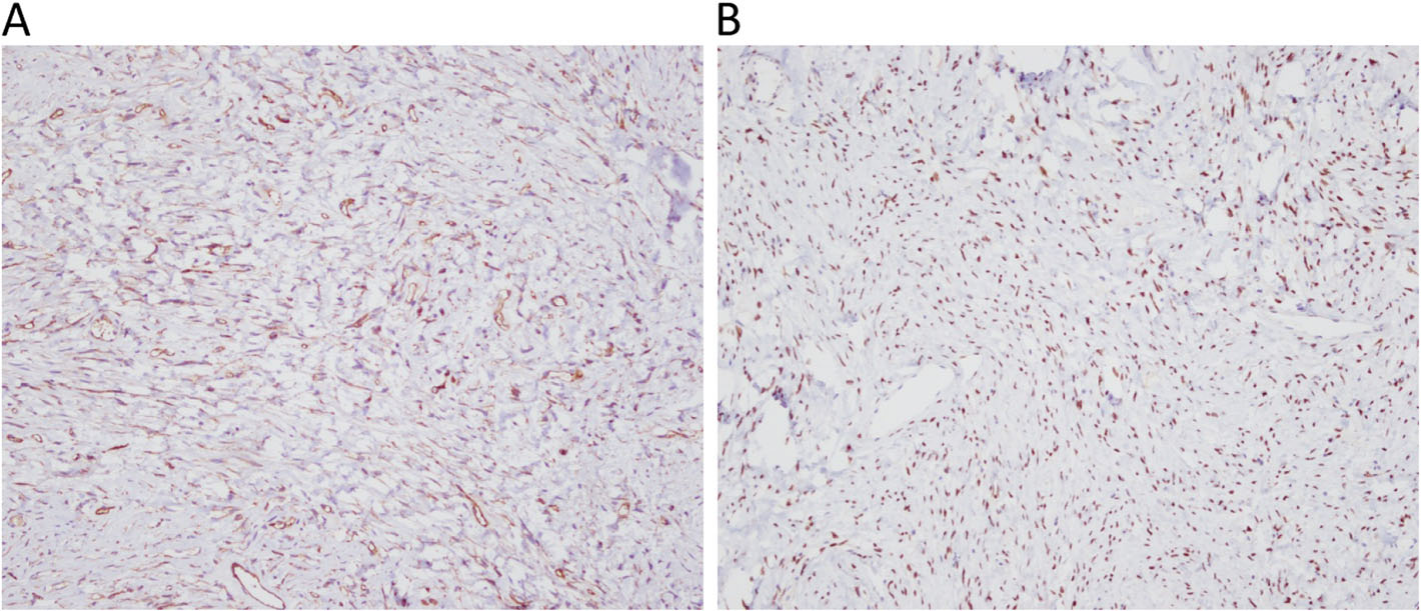
Immunohistochemical features. (**a**) Positive nuclear expression of STAT6 in tumor cells. (**b**) Positive expression of CD34 in tumor cells

The resected specimen consisted of three lobulated and contiguous firm tumors, measuring 7.5 × 6.3 × 3.8 cm in size and weighing 76.5 g in total (Fig. 2a). Cut section of the tumor showed a well-defined, lobulated, whitish and firm tumor with some mucinous components (Fig. 2b). Microscopically, it was a hyper-cellular tumor with a vaguely fascicular growth pattern forming a patternless growth architecture (Fig. 3a) with minimal nulcear atypia rate and a mitotic count < 4 per 10 high power field. A few thin-walled, branching “staghorn appearance” vessels were also present in the tumor (Fig. 3b). The tumor cells had an ovoid to short spindle shape with indistinct borders and dispersed chromatin within vesicular nuclei. Immunohistochemically, the tumor cells were positive for STAT-6 (nuclear expression) (Fig. 4a) and CD34 (Fig. 4b), and negative for actin, desmin, CD117 and DOG-1. Based on the morphology and immunohistochemical studies, the diagnosis of SFT was made. The patient was alive without tumor recurrence or distant metastasis during ongoing follow-up for 9 months post-operatively with semi-annual CTs.

## Discussion and conclusions

SFT is a soft tissue tumor which usually presents as a firm, grey-to-white colored, well-circumscribed solid mass. It was first reported by Klemperer and Rabin in 1931 in the pleura. Although the disease most commonly occurs in the pleura, extra-thoracic SFTs have been reported in many sites including the head and neck [10], intracranial and spinal cord meninges [11], eyes [12], thyroid [13], larynx [14], gastrointestinal system [15–17], genitourinary tract [18–20], pelvis [21] and soft tissue [22]. The most common extra-pleural locations are the meninges, followed by subcutaneous tissues of the lower limbs, the retroperitoneum, and the orbit [23]. Extra-pleural SFTs share similar histological features with pleural SFTs [23].

There are very few reported cases of genitourinary SFTs, and SFTs of the scrotum or para-testicular SFTs are extremely rare (Table 1). This is only the fifth report of SFT of the scrotum in the English medical literature, and the only report with three documented connected nodules, which is different from the usual appearance of a SFT with a single solitary nodule.

**Table 1:** Reported cases of paratesticular SFTs

Reference	Year	Age	Initial presentation	Location	Tumor size (cm)	Treatment	Recurrence/ Follow-up time	Immuno-histochemical features
Marquez MA et al. [2]	2001	67	Paratesticular mass	NA	9	Surgical excision	NA	CD34+, Vimentin+, Actin-, S100-, Keratin-
Garcia TM et al. [3]	2006	22	Pain	Left tunica vaginalis testis	3	Surgical excision with intraoperative biopsy	None/12 months	NA
Gutierrez-Diaz CM et al. [5]	2011	53	NA	Paratesticular	NA	NA	NA	CD34+, BCL-2 +, vimentin+
Lee GE et al. [4]	2011	61	Slow growing mass	Left scrotal sac	5 × 4	Surgical excision	NA/NA	CD34+
Barazani Y et al. [6]	2012	26	Painless firm mass	Left scrotum	6.1 × 5.5 × 4.3	Inguinal exploration	None/NA	CD34+, BCL-2 +, SMA -, Desmin-, S100-
Hu SB et al. [7]	2014	31	Left inguinoscrotal swelling	Left spermatic cord	3 × 2	Inguinal exploration	None/25 months	CD99+, Bcl-2+, Partial CD34+,, Focal S-100+, SMA+, CD68-
Zhou YH et al. [8]	2015	61	Slow growing mass	Left scrotum	4 × 4.5 × 5	Surgical excision (Inguinal)	None/6 months	CD34+, CD99+, Vimentin+, CD117, S100-, SMA-, Desmin-, CD68-
Zhao XY et al. [9]	2017	77	Painless mass	Left scrotum	11x9x8	Surgical excision	None/18 months	CD34+, CD99+, STAT6+

*NA* not available, *CD* cluster of differentiation, STAT6, activator of transcription 6

The diagnosis of extra-pleural SFTs is challenging and relies on its clinical manifestations or imaging studies. Differential diagnosis of SFTs arising from para-testicles soft tissues can be challanging since it can show great similarity to those spindle cell fibroblastic associated tumors, such as angiomyolipomas, leimyoma, fibrosarcomas and gastrointestinal stromal tumors (GIST). Therefore, immunohistochemistry plays an important role in the diagnosis of SFTs. Traditionally, CD34 and BLC-2 have a high sensitivity for SFTs, and CD34 is only absent in 5 to 10% of typical SFTs [24]. BCL-2 is seen in almost all cases of SFTs [25, 26]. However, these traditional markers are not specific to SFTs, and they may also be present in many other mesenchymal tumors mimicking SFTs, which may lead to confusion and uncertainty in the diagnosis. In 2013, Chmelecki et al. [27] and Robinson et al. [28] reported that NAB2-STAT6, a new fusion gene expression present in the vast majority of SFTs, can be used as a unique molecular marker for the diagnosis of SFTs. Subsequent reports have demonstrated that the immunohistochemical nuclear expression of STAT6 in SFTs can distinguish SFTs from other histologic mimics, and that it can be used as a diagnostic tool [29–31]. Thus, in current clinical settings, the diagnosis of SFTs can be made if STAT6 nuclear expression, CD34 and BCL-2 are all strongly positive. NAB2-STAT6 fusion genetic tests may not be necessary for the diagnosis of SFTs under such circumstances.

SFTs are usually considered to follow a benign clinical course with a low potential of malignant change, and surgical excision of the lesion is usually sufficient. There were no current consensus on how the surgical approach should be done. Since SFTs usually presents as a low-malignant potential tumor, both inguinal and scrotal approach for tumor exploration and excional had been reported. However, malignant pathology and behavior have been described in about 20% of SFTs [32]. The diagnosis of malignant SFT is based on pathologic examinations of histological features. The presence of hypercellularity, infiltrative margin growth, > 4 mitotic counts per 10 high-power fields and nuclear atypia have been reported to be histological malignant components [29, 32, 33]. In a multicenter study including 81 patients with surgically treated SFTs, patients with histologically malignant SFTs had higher local recurrence rates and higher incidence of metastasis [34]. Positive surgical margins, tumor size > 10 cm, and the presence of a high mitotic rate have been reported to be significantly correlated with a higher incidence of metastasis and worse overall survival.

In conclusion, SFT is a rare mesenchymal spindle cell neoplasm originally reported in the pleura, but it can be found in various sites throughout the body. Scrotal SFTs are very rare and only five cases have been reported to date. Treatment often involves surgical resection, and a definite diagnosis is made with the help of immunohistochemistry, especially the nuclear expression of STAT6. SFTs are often benign but there is a slight chance of malignancy. Patients with histologically malignant features may have worse prognosis. The current general consensus for the management of SFTs is long-term follow-up after surgical excision of the tumor. In our experience, we performed surgcial resection of the tumor via scrotal approach without additional interventions. 6-month follow-up with CT scan was conducted, we will continue the follow-up of this case to monitor long-term outcome of this rare disease.

## Data Availability

Records and data pertaining to this case are in the patient’s secure medical records in Mackay Memorial Hospital.

## Abbreviation

CT: Computed tomography
SFT: Solitary fibrous tumor

## References

[1] Klemperer P, Coleman BR (1992). Primary neoplasms of the pleura. A report of five cases. Am J Ind Med.

[2] Márquez AM, Vicioso LR, Castro AL, Casals JS, Alcázar JR, Matilla AV (2001). Paratesticular solitary fibrous tumor. J Archivos Espanoles de Urologia.

[3] García MT, Beltrán JA, Santolaya IG, Carrascosa VL, Tarín MP, CJDLJAedu S (2006). Solitary fibrous tumor of the tunica vaginalis testis. Arch Esp Urol.

[4] Lee GE, Rha SE, Byun JY, Lee K, SWJJoUiM K (2011). Paratesticular solitary fibrous tumor: a rare cause of a hypervascular extratesticular mass. J Ultrasound Med.

[5] Ceballos MEG-D, Hernández-Solís A, Cruz-Ortiz H, González-Atencio Y, Cicero-Sabido RJCC (2011). Solitary fibrous tumor: clinicopathological study of 16 cases. Modern Pathology : an official journal of the United States and Canadian Academy of Pathology, Inc.

[6] Barazani Y, Tareen B (2012). Rare case of paratesticular solitary fibrous tumour (lipomatous hemangiopericytoma). Can Urol Assoc J.

[7] Hu S, Yi L, Yang L, Wang Y (2015). Solitary fibrous tumor of the spermatic cord: a case report and literature review. Exp Ther Med.

[8] Zhou Y, Gong G, Tang Y, Tang J, Gan Y, YJIjoc D (2015). Pathology e: Paratesticular solitary fibrous tumor: a case report and review of literature. Int J Clin Exp Pathol.

[9] Zhao XY, Zeng M, Yang QY, Jing CP, Zhang Y (2017). Scrotum solitary fibrous tumor: a case report and review of literature. Medicine (Baltimore).

[10] Smith SC, Gooding WE, Elkins M, Patel RM, Harms PW, McDaniel AS, Palanisamy N, Uram-Tuculescu C, Balzer BB, Lucas DR (2017). Solitary fibrous tumors of the head and neck: a multi-institutional Clinicopathologic study. Am J Surg Pathol.

[11] Bouvier C, Métellus P, de Paula AM, Vasiljevic A, Jouvet A, Guyotat J, Mokhtari K, Varlet P, Dufour H, Figarella-Branger DJBP (2012). Solitary fibrous tumors and hemangiopericytomas of the meninges: overlapping pathological features and common prognostic factors suggest the same spectrum of tumors. J Brain Pathology.

[12] Petrovic A, Oberic A, Moulin A, Hamedani M (2015). Ocular adnexal (orbital) solitary fibrous tumor: nuclear STAT6 expression and literature review. Graefe's Arch Clin Exp Ophthalmol = Albrecht von Graefes Archiv fur klinische und experimentelle Ophthalmologie.

[13] Ghasemi-Rad M, Wang KY, Jain S, Lincoln CM (2019). Solitary fibrous tumor of thyroid: a case report with review of literature. Clin Imaging.

[14] Dotto JE, Ahrens W, Lesnik DJ, Kowalski D, Sasaki C, Flynn S (2006). Solitary fibrous tumor of the larynx: a case report and review of the literature. Arch Pathol Lab Med.

[15] Urabe M, Yamagata Y, Aikou S, Mori K, Yamashita H, Nomura S, Shibahara J, Fukayama M, Seto Y (2015). Solitary fibrous tumor of the greater omentum, mimicking gastrointestinal stromal tumor of the small intestine: a case report. Int Surg.

[16] Bratton L, Salloum R, Cao W, Huber AR (2016). Solitary fibrous tumor of the sigmoid Colon masquerading as an adnexal neoplasm. Case Rep Pathol.

[17] D'Amico FE, Ruffolo C, Romano M, DID M, Sbaraglia M, Dei Tos AP, Garofalo T, Giordano A, Bassi I, Massani M (2017). Rare neoplasm mimicking Neuoroendocrine pancreatic tumor: a case report of solitary fibrous tumor with review of the literature. Anticancer Res.

[18] Abeygunasekera AM, Ginige AP, Liyanage IS, Hareendra K (2015). A solitary fibrous tumor of the kidney. J Cancer Res Ther.

[19] Kouba E, Simper NB, Chen S, Williamson SR, Grignon DJ, Eble JN, MacLennan GT, Montironi R, Lopez-Beltran A, Osunkoya AO (2017). Solitary fibrous tumour of the genitourinary tract: a clinicopathological study of 11 cases and their association with the NAB2-STAT6 fusion gene. J Clin Pathol.

[20] Prunty MC, Gaballah A, Ellis L, Murray KS (2018). Solitary fibrous tumor of the pelvis involving the urinary bladder. Urology.

[21] Fernandez A, Conrad M, Gill RM, Choi WT, Kumar V, Behr S (2018). Solitary fibrous tumor in the abdomen and pelvis: a case series with radiological findings and treatment recommendations. Clin Imaging.

[22] Nielsen GP, O'Connell JX, Dickersin GR, Rosenberg AE (1997). Solitary fibrous tumor of soft tissue: a report of 15 cases, including 5 malignant examples with light microscopic, immunohistochemical, and ultrastructural data. Mod Pathol: an official journal of the United States and Canadian Academy of Pathology, Inc.

[23] Ronchi A, Cozzolino I, Zito Marino F, Accardo M, Montella M, Panarese I, Roccuzzo G, Toni G, Franco R, De Chiara A (2018). Extrapleural solitary fibrous tumor: a distinct entity from pleural solitary fibrous tumor. An update on clinical, molecular and diagnostic features. Ann Diagn Pathol.

[24] Chan JK (1997). Solitary fibrous tumour--everywhere, and a diagnosis in vogue. Histopathology.

[25] Chilosi M, Facchettti F, Dei Tos AP, Lestani M, Morassi ML, Martignoni G, Sorio C, Benedetti A, Morelli L, Doglioni C (1997). bcl-2 expression in pleural and extrapleural solitary fibrous tumours. J Pathol.

[26] Suster S, Fisher C, Moran CA (1998). Expression of bcl-2 oncoprotein in benign and malignant spindle cell tumors of soft tissue, skin, serosal surfaces, and gastrointestinal tract. Am J Surg Pathol.

[27] Chmielecki J, Crago AM, Rosenberg M, O'Connor R, Walker SR, Ambrogio L, Auclair D, McKenna A, Heinrich MC, Frank DA (2013). Whole-exome sequencing identifies a recurrent NAB2-STAT6 fusion in solitary fibrous tumors. Nat Genet.

[28] Robinson DR, Wu YM, Kalyana-Sundaram S, Cao X, Lonigro RJ, Sung YS, Chen CL, Zhang L, Wang R, Su F (2013). Identification of recurrent NAB2-STAT6 gene fusions in solitary fibrous tumor by integrative sequencing. Nat Genet.

[29] Yoshida A, Tsuta K, Ohno M, Yoshida M, Narita Y, Kawai A, Asamura H, RJTAjosp K (2014). STAT6 immunohistochemistry is helpful in the diagnosis of solitary fibrous tumors. Am J Surg Pathol.

[30] Doyle LA, Vivero M, Fletcher CD, Mertens F, Hornick JLJMP (2014). Nuclear expression of STAT6 distinguishes solitary fibrous tumor from histologic mimics. Mod Pathol.

[31] Zhang X, Cheng H, Bao Y, Tang F, Wang Y (2016). diagnostic value of STAT6 immunohistochemistry in solitary fibrous tumor/meningeal hemangiopericytoma. Zhonghua bing li xue za zhi = Chinese J Pathol.

[32] Gold JS, Antonescu CR, Hajdu C, Ferrone CR, Hussain M, Lewis JJ, Brennan MF, Coit DG (2002). Clinicopathologic correlates of solitary fibrous tumors. Cancer.

[33] England DM, Hochholzer L, McCarthy MJ (1989). Localized benign and malignant fibrous tumors of the pleura. A clinicopathologic review of 223 cases. Am J Surg Pathol.

[34] van Houdt WJ, Westerveld CM, Vrijenhoek JE, van Gorp J, van Coevorden F, Verhoef C, van Dalen T (2013). Prognosis of solitary fibrous tumors: a multicenter study. Ann Surg Oncol.

